# MicroRNA-146a modulates B-cell oncogenesis by regulating Egr1

**DOI:** 10.18632/oncotarget.3433

**Published:** 2015-04-13

**Authors:** Jorge R. Contreras, Jayanth Kumar Palanichamy, Tiffany M. Tran, Thilini R. Fernando, Norma I. Rodriguez-Malave, Neha Goswami, Valerie A. Arboleda, David Casero, Dinesh S. Rao

**Affiliations:** ^1^ Department of Pathology and Laboratory Medicine, UCLA, Los Angeles, CA USA; ^2^ Cellular and Molecular Pathology Ph.D. Program, UCLA, Los Angeles, CA USA; ^3^ Jonsson Comprehensive Cancer Center, UCLA, Los Angeles, CA USA; ^4^ Broad Stem Cell Research Center, UCLA, Los Angeles, CA USA

**Keywords:** microRNA, B-cell, lymphoma, leukemia, c-Myc

## Abstract

miR-146a is a NF-κB induced microRNA that serves as a feedback regulator of this critical pathway. In mice, deficiency of miR-146a results in hematolymphoid cancer at advanced ages as a consequence of constitutive NF-κB activity. In this study, we queried whether the deficiency of miR-146a contributes to B-cell oncogenesis. Combining miR-146a deficiency with transgenic expression of c-Myc led to the development of highly aggressive B-cell malignancies. Mice transgenic for c-Myc and deficient for miR-146a were characterized by significantly shortened survival, increased lymph node involvement, differential involvement of the spleen and a mature B-cell phenotype. High-throughput sequencing of the tumors revealed significant dysregulation of approximately 250 genes. Amongst these, the transcription factor Egr1 was consistently upregulated in mice deficient for miR-146a. Interestingly, transcriptional targets of Egr1 were enriched in both the high-throughput dataset and in a larger set of miR-146a-deficient tumors. miR-146a overexpression led to downregulation of Egr1 and downstream targets with concomitant decrease in cell growth. Direct targeting of the human EGR1 by miR-146a was seen by luciferase assay. Together our findings illuminate a bona fide role for miR-146a in the modulation of B-cell oncogenesis and reveal the importance of understanding microRNA function in a cell- and disease-specific context.

## INTRODUCTION

MicroRNAs (miRNAs) are a class of small non-coding RNAs, 21–22 nucleotides in length, which have physiological roles in many developmental systems [1]. miRNAs primarily act through post-transcriptional repression of target mRNAs via short complementary sequences in the 3′untranslated region (UTR) of mRNA transcripts [2, 3]. It has been reported that nearly 2000 miRNAs exist in the human genome and more than half of protein-coding genes are potential targets for miRNAs [4]. Both oncogenic and tumor suppressive miRNAs have been described in oncogenesis, acting via repression of tumor-suppressive and growth-promoting targets, respectively [5–8]. It is important to note, however, that miRNA regulation of gene expression is highly context-dependent: they regulate a cell type-specific transcriptome generated by a set of oncogenic or developmental transcriptional regulators. Hence, uncovering the oncogenic role of a miRNA requires the study of lineage specific transcriptional dysregulation.

miR-146a was discovered as a transcriptional target of the NF-κB pathway acting as a negative feedback regulator of this pathway and repressing some key components, such as *Traf6* and *Irak1* [9–13]. In line with its function in the NF-κB pathway, miR-146a deficiency in mice results in the development of a hyper inflammatory phenotype characterized by myeloid proliferation, lymphoid hyperplasia, T-cell hyper activation and autoantibody production [9, 11, 14, 15]. Subsequently, aged knockout mice develop myeloid and lymphoid malignancies [9, 11]. These phenotypes are characterized by a dependence on constitutive NF-κB activity, as demonstrated by the correction of many phenotypes by deletion of elements of NF-κB signaling or downstream mediators [11, 16].

Constitutive NF-κB activity is a hallmark of many different types of cancer including B-cell malignancies [17]. The activated B-cell type of diffuse large B-cell lymphoma (ABC-DLBCL), which demonstrates constitutive NF-κB activation, is more aggressive and leads to worse outcomes in patients. Currently, several components of the NF-κB pathway have been found mutated in DLBCL, producing activation of NF-κB [18, 19]. The role of miR-146a as a negative regulator of this critical pathway, along with the development of B-cell malignancies in knockout mice, suggest that loss of miR-146a via undefined mechanisms may represent a pathogenetic event in B-cell malignancies that contributes to constitutive NF-κB activity.

In addition to being regulated by NF-κB, miR-146a has been shown to be positively regulated by the potent oncogene, *c-Myc*, in a melanoma cell line [20]. In contrast, primary samples of B-cell lymphoma with high levels of *c-Myc* expression show dramatic downregulation of miR-146a expression, and additional studies demonstrate negative regulation of miR-146a by c-Myc [21–23]. This led us to question the role that miR-146a plays in *c-Myc*-mediated oncogenesis in the B-cell lineage. Since *c-Myc* is a powerful transcriptional regulator with a specific transcriptome, we hypothesized that miR-146a mediated effects on the *c-Myc* gene expression program would reveal unique cancer relevant pathways. To test our hypotheses, we intercrossed the Eμ-Myc mouse with miR-146a-deficient animals. We found that miR-146a deficiency accelerates oncogenesis, decreases survival, and alters the differentiation stage of the tumors that are formed in the resulting mice. Histopathologic and flow cytometric analyses revealed a distinctive pattern of involvement in miR-146a-deficient animals. Mechanistically, few genes were significantly differentially regulated between wild-type and miR-146a-deficient, *c-Myc* driven tumors. Of these, *Egr1* and its downstream mediators were identified as a novel pathway regulated by miR-146a in B-cells. Our findings promise to open up a new area of research and demonstrate a tumor suppressive function for miR-146a in B-cell oncogenesis.

## RESULTS

### miR-146a deficiency decreases survival of Eμ-Myc transgenic mice

**Figure 1:**
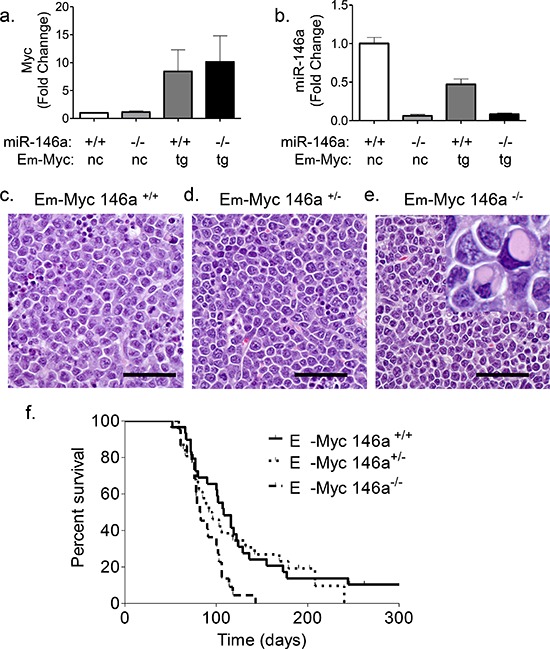
miR-146a deficiency causes increased mortality in Eμ-Myc mice **a.** RT-qPCR for c-Myc was performed on splenic B-cells from WT and miR-146a^−/−^ mice or tumor samples from Eμ-Myc miR-146a^+/+^ and Eμ-Myc miR-146a^−/−^ animals (*n* = 3, 3, 12, 11 respectively) (nc: non-carrier; tg: transgene). **b.** RT-qPCR for miR-146a was performed from the same samples as for c-Myc. **c–e.** Hematoxylin and Eosin (H&E) stained sections of lymph node tumors derived from Eμ-Myc miR-146a^+/+^, Eμ-Myc miR-146a^+/−^ and Eμ-Myc miR-146a^−/−^mice, respectively. The inset in (e) shows a subset of cells with immunoglobulin concretions. Scale bar for Figures 1c-e, 40 μm. **f.** Kaplan Meier survival curve of mice with Eμ-Myc oncogene and wild-type, heterozygous, or homozygous deficiency of miR-146a (*n* = 26 for Eμ-Myc miR-146a^+/+^(Solid line in graph), *n* = 23 for Eμ-Myc miR-146a^+/−^ (Dotted line on graph), *n* = 22 for Eμ-Myc miR-146a^−/−^ (Dashed line on graph);w.t. vs. het comparison, Log-Rank Test, *p* = 0.6725; w.t. vs. k.o. comparison, Log-Rank Test, *p* = 0.0027).

Given the proposed roles for miR-146a in tumor suppression and negative feedback regulation of the NF-κB pathway, we examined whether miR-146a deficiency would synergize with *c-Myc* during B-cell oncogenesis. miR-146a-deficient and Eμ-Myc transgenic mice were bred to yield cohorts of mice that carried the Eμ-Myc transgene with wild-type, heterozygous or homozygous knockout alleles of miR-146a (Figure 1a–1b). Most tumors that formed in Eμ-Myc mice showed a lymphoblastic morphology with numerous mitotic figures and apoptotic bodies on H&E sections (Figure 1c–1e). Conversely, tumors from the miR-146a-deficient mice demonstrated a more heterogeneous appearance. Many tumors had lymphoblastic morphology, but others showed a plasmacytoid appearance, including eosinophilic cytoplasmic concretions, suggestive of immunoglobulin deposits (Figure 1e inset shows cells with immunoglobulin concretions). Eμ-Myc miR-146a^+/−^ mice did not have a significant reduction in their survival (Figure 1f). On the other hand, homozygous deficiency caused a decrease in survival from 104.5 days to 82.5 days (Figure 1f). Gender differences were noted, with female miR-146a–^/–^ mice showing significant differences in survival, while males only showed a trend towards reduced survival (Supplementary Figure 1a–1d). Finally, virtually all mortality in both sets of mice was attributable to tumor formation (data not shown).

### miR-146a-deficient tumors demonstrate differential anatomic patterns of involvement

**Figure 2:**
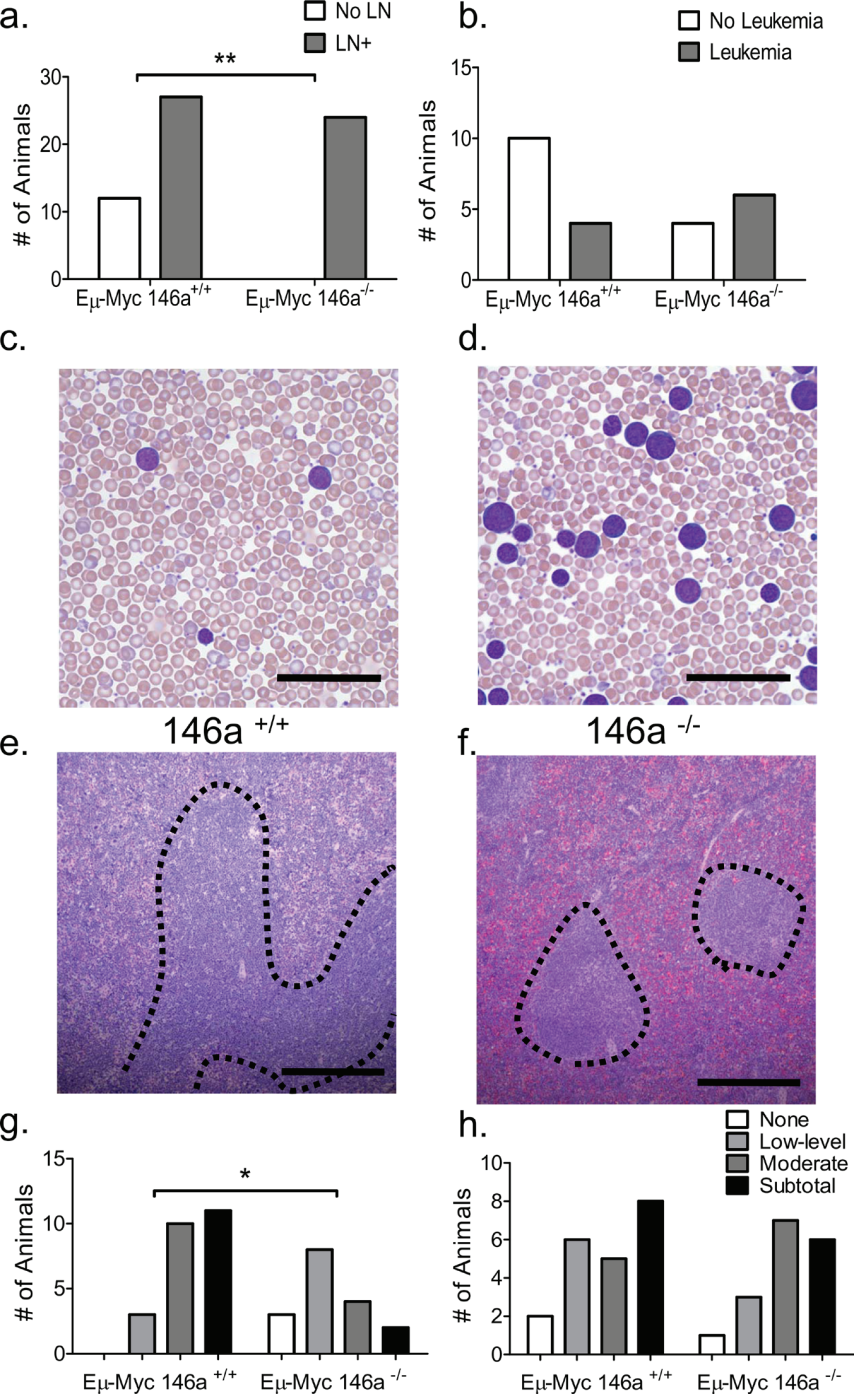
miR-146a-deficient tumors show a differential pattern of anatomic involvement **a.** Macroscopic lymph node involvement is significantly increased in miR-146a^−/−^ deficient mice (*n* = 39 for Eμ-Myc miR-146a^+/+^ and *n* = 24 for Eμ-Myc miR-146a^−/−^; Fisher's exact test, *p* = 0.002). **b.** Quantitation of the incidence of leukemia, defined as a peripheral white blood cell count of greater than 30, 000/μL, in these mice, shows a trend towards statistical significance between the groups (*n* = 14 for Eμ-Myc miR-146a^+/+^ and *n* = 10 for Eμ-Myc miR-146a^−/−^; Fisher's exact test, *p* = 0.21). **c–d.** Wright stained peripheral blood smears from mice with miR-146a-sufficient and deficient Eμ-Myc driven tumors. Scale bar, 40 μm. **e.** H&E stained section of Eμ-Myc miR-146^+/+^spleen, low power image. Dotted lines delineate expanded white pulp. Scale bar, 400 μm. **f.** Low power view of an H&E stained section showing relative sparing of the white pulp (dotted lines) in Eμ-Myc miR-146a^−/−^ mice. Scale bar, 400 μm. **g.** Quantitation of splenic white pulp involvement in Eμ-Myc miR-146a^+/+^ and Eμ-Myc miR-146a^−/−^ mice on an ordinal 4-point scale going from no involvement to subtotal involvement of the spleen (*n* = 24 for Eμ-Myc miR-146a^+/+^*n* = 17 for Eμ-Myc miR-146a^−/−^; Chi Square Test, *p* = 0.004). **h.** Quantitation of splenic red pulp involvement in Eμ-Myc miR-146a^+/+^ and Eμ-Myc miR-146a^−/−^ mice on an ordinal 4-point scale going from no involvement to subtotal involvement of the spleen (*n* = 24 for Eμ-Myc miR-146a^+/+^*n* = 17 for Eμ-Myc miR-146a^−/−^; Chi Square Test, *p* = 0.671).

Anatomically, tumors in both sets of mice showed differential patterns of involvement of hematopoietic and lymphoid organs, with virtually all mice showing thymic involvement. 31% of Eμ-Myc miR-146a^+/+^ did not show any lymph node involvement, whereas all of the miR-146a^−/−^ did show involvement (Figure 2a). While the majority of mice in both groups showed small numbers of circulating tumor cells in the peripheral blood, 6/10 Eμ-Myc miR-146a^−/−^ mice examined showed frank leukemia (defined as a white blood cell count of greater than 30,000/μL) (Figure 2b–2d). This was in contrast to the lower numbers of Eμ-Myc miR-146a^+/+^ mice that demonstrated leukemia by blood counts (4/14). Amongst mice with predominantly solid tumors, miR-146a deficiency caused a statistically significant increase in peripheral blood CD11b+ myeloid cells but not in B220+ B-cells, CD3ε+ T-cells, hemoglobin or platelets (Supplementary Figure 2a–2f). This may represent the propensity of miR-146a-deficient hematopoietic progenitors to produce increased numbers of myeloid cells. Bone marrow analysis of these mice found similar proportions of myeloid cells, erythroid cells, and B- lymphocytes (Supplementary Figure 2g–2i).

Mice in both groups demonstrated enlarged spleens, with average weights of approximately 400 mg (Supplementary Figure 3a). In Eμ-Myc miR-146a^+/+^ mice there was involvement of the white pulp with contiguous spread between the lymphoid follicles (Figure 2e, dotted area). High power views showed the malignant cells in both the white and red pulp (Supplementary Figure 3b–3c). On the other hand, Eμ-Myc miR-146a^−/−^ mice showed extensive involvement of the red pulp with relative sparing of the white pulp (Figure 2f). Using a semi-quantitative 4-point scale to grade involvement, we found that white pulp involvement was significantly higher in Eμ-Myc miR-146a^+/+^ mice compared to knockout mice (Figure 2g). Red pulp involvement was not different between the two groups (Figure 2h). Despite these differential patterns of involvement, the relative numbers of B-cells, T-cells and myeloid cells in the spleen were equivalent between the two groups of mice (Supplementary Figure 3d–3k). Together, the data suggest similar overall infiltration of the spleen (given similar weights and cellular composition), but a predilection for the red pulp when miR-146a is deficient, suggesting that the deficiency of miR-146a may change the homing properties of the malignant B-cells. The patterns of involvement are somewhat reminiscent of certain subtypes of B-cell lymphoma/leukemia that show peripheral blood involvement and red pulp involvement in the spleen, but are not correlated with NF-κB activity or histologic subtype in humans.

### miR-146a-deficient tumors demonstrate a mature B-cell phenotype

**Figure 3:**
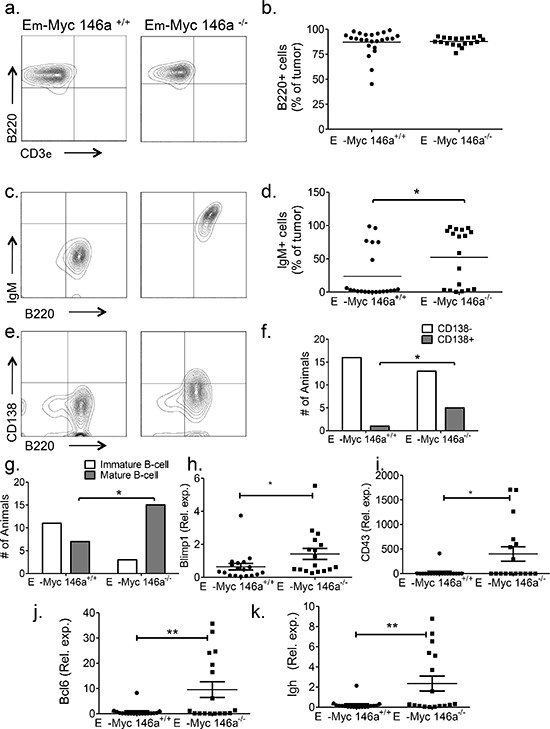
miR-146a deficiency causes a mature B-cell phenotype in Eμ-Myc mice **a.** Representative FACS plot showing staining for B220 and CD3ε in Eμ-Myc miR-146a^+/+^ and Eμ-Myc miR-146a^−/−^mice. **b.** FACS shows that both groups of mice demonstrate B-cell tumors (*n* = 24 for Eμ-Myc miR-146a^+/+^ and *n* = 19 for Eμ-Myc miR-146a^−/−^; *t*-test *p* = 0.794). **c.** Representative FACS plots stained for B220 and IgM from tumors derived from Eμ-Myc miR-146a^+/+^ and Eμ-Myc miR-146a^−/−^ mice. **d.** Percentage of IgM positive cells in tumors from both cohorts showing increased IgM positivity in Eμ-Myc miR-146a^−/−^ tumors (*n* = 25 for Eμ-Myc miR-146a^+/+^ and *n* = 17 for Eμ-Myc miR-146a^−/−^; *t*-test, *p* = 0.03). **e.** Representative FACS plots stained for B220 and CD138 from tumors derived from Eμ-Myc miR-146a^+/+^ and Eμ-Myc miR-146a^−/−^ mice. **f.** Dichotomized CD138 expression data (see methods for details on dichotomization), showing animals with CD138+ versus CD138- tumors (*n* = 18 for Eμ-Myc miR-146a^+/+^ and *n* = 18 for Eμ-Myc miR-146a^−/−^; Chi-square test, one-sided *p* = 0.04). **g.** Tumors were dichotomized as being either immature (double negative for CD138 and IgM) or mature (having expression of either marker) (*n* = 18 for Eμ-Myc miR-146a^+/+^ and *n* = 18 for Eμ-Myc miR-146a^−/−^; Chi-square test, *p* = 0.0062). **h–k.** RT-qPCR data for Blimp1, CD43, Bcl6, and Ighδ in tumors from Eμ-Myc miR-146a^+/+^ (*n* = 18)and Eμ-Myc miR-146a^−/−^ mice (*n* = 17). All comparisons showed statistically significant differences by *T*-test (*p* = 0.05 for Blimp1 (h), *p* = 0.014 for CD43 (i), *p* = 0.0070 for Bcl6 (j), and *p* = 0.0067 for Ighδ(k).

To further characterize the increased mortality seen in the miR-146a-deficient mice, we undertook immunophenotypic analyses. The tumors in both sets of mice were predominantly of B-cell phenotype (Figure 3a–3b). Similarly, Eμ-Myc mice with a heterozygous deficiency for miR-146a also developed B-cell tumors (Supplementary Figure 4a–4d). To examine the stage of differentiation, we examined expression of IgM, finding that greater than 70% of tumors from Eμ-Myc miR-146a^+/+^ mice were IgM-. In contrast, only 42% of tumors from Eμ-Myc miR-146a^−/−^ mice were IgM- (Figure 3c–3d). Amongst IgM- tumors, several were plasmacytic, shown by morphology and staining for CD138 (Figure 3e). Next, we dichotomized the data by mean fluorescent intensity (MFI), finding that CD138+ tumors were more frequently seen in the miR-146a-deficient background (Figure 3f). When we combined positivity for CD138 and IgM, most miR-146a-deficient tumors showed a mature B-cell phenotype (either IgM+ or CD138+) whereas miR-146a-sufficient tumors were negative for both IgM and CD138 (Figure 3). Tumor cells from miR-146a-deficient mice showed lower expression of memory B-cell/activation related antigen, CD80 (Supplementary Figure 4e–4f), but similar expression of CD44 (Supplementary Figure 4g–4h). To further characterize the stage of B-cell differentiation in these tumors, we performed RT-qPCR to quantitate the expression of genes involved in B-cell differentiation. We found that transcripts for Blimp1, CD43, Bcl6 and Ighδ were all more highly expressed in tumors from miR-146a-deficient mice (Figure 3h–3k). Interestingly, female mice, which showed a statistically significant difference in survival, showed similar trends in their immunophenotypic profiles (Supplementary Figure 5a–5f) as well as in gene expression of maturation-related B-cell transcripts (Supplementary Figure 5g–5j) when compared to the group overall. Together these findings indicate that miR-146a-deficient tumors are composed of malignant B-cells that derive from a different stage of differentiation than tumors sufficient for miR-146a.

### miR-146a-deficient tumors show a limited difference in transcriptome expression, including many putative targets of miR-146a

**Figure 4:**
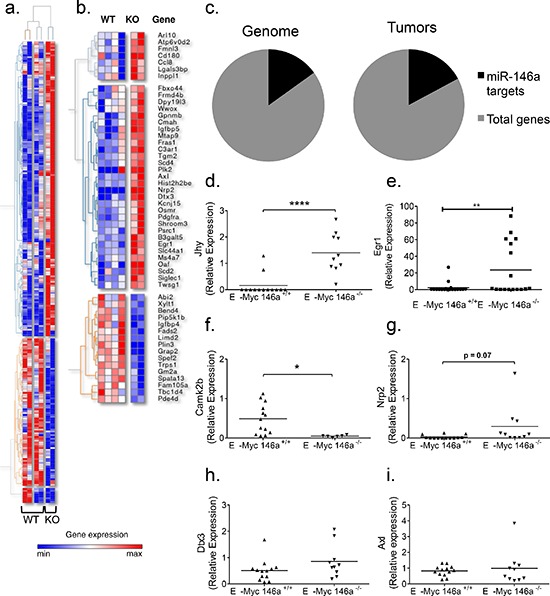
Gene expression analyses of Eμ-Myc driven tumors with miR-146a deficiency **a.** Genes differentially expressed between Eμ-Myc miR-146a^+/+^ and Eμ-Myc miR-146a^−/−^ tumors. **b.** Differentially expressed genes with miR-146a sites in their UTR as predicted by the TargetScan algorithm. The heat map color scale represents, for each gene, the relative expression level using the average mean gene expression as a reference. **c.** Graphical representation of the percentage of the genome (left) predicted to be targeted by miR-146a (2773 predicted targets out of 18, 393 annotated UTRs), compared with the percentage of upregulated genes in the tumor dataset (right) that are predicted miR-146a targets (29 predicted targets out of 169 upregulated genes). No statistically significant enrichment was found (Chi-square test). **d–i.** RT-qPCR of genes found to be differentially regulated by RNA-sequencing analysis, including *Jhy* (d; *t*-test, *p* < 0.0001), *Egr1* (e; *t*-test, *p* = 0.0086), *Camk2b* (f; *t*-test, *p* = 0.01), *Nrp2* (g; *t*-test, *p* = 0.0743), *Dtx3* (h; *t*-test, *p* = 0.127) and *Axl* (i; *t*-test, *p* = 0.616). The three genes on the left (*Jhy, Camk2b and Dtx3*) were amongst the most differentially regulated genes between the miR-146a sufficient and deficient tumors by RNA sequencing. The three genes on the right (*Egr1, Nrp2 and Axl*) represent putative targets of miR-146a (For RT-qPCR analysis *n* = 13 for Eμ-Myc miR-146a^+/+^ and *n* = 10 for Eμ-Myc miR-146a^−/−^ for panels d, g, h, i; *n* = 13 for Eμ-Myc miR-146a^+/+^ and *n* = 6 for Eμ-Myc miR-146a^−/−^ for panel f; *n* = 18 for Eμ-Myc miR-146a^+/+^ and *n* = 17 for Eμ-Myc miR-146a^−/−^ for panel e).

To define a mechanistic basis for miR-146a-deficient B-lymphomagenesis, we performed RNA-sequencing on four miR-146a sufficient and two miR-146a-deficient tumors. Based on this comparison, we arrived at a list of 249 genes that were differentially regulated with an adjusted *p*-value of 0.05 or lower (Figure 4a). We then searched the dataset for miR-146a targets predicted by TargetScan [2, 3]. Of the differentially regulated genes, 53 genes are predicted to be miR-146a targets (Figure 4b). When we examined the genes that were upregulated, 29 out of 140 genes were predicted miR-146a targets (Figure 4c), and this did not represent a statistical enrichment. Next, we confirmed some of the findings by RT-qPCR in the larger set of tumor samples that we had collected. Four of the top ten genes from RNA sequencing had significantly different expression levels in the tumors when assayed by qPCR. These genes include *Jhy* and *Camk2b* (Figure 4d and 4f). *Jhy* is a recently described novel gene with no known function in oncogenesis or hematopoiesis; while *Camk2b* has a previously described putative role in epithelial cancer [24]. The gene *Dtx3*, which showed differential regulation by RNA-sequencing, was not differentially expressed in the larger set of tumor samples (Figure 4h). Other genes that were differentially expressed included the putative target *Egr1*, with *Nrp2* showing a trend towards differential expression (Figure 4e and 4g). A third predicted target, *Axl*, failed to show differential regulation by qPCR in this larger set of samples (Figure 4i). Hence, the transcriptome data provided us with a starting point for understanding tumorigenesis, uncovering putative miR-146a targets in the setting of B-cell oncogenesis.

### The transcriptome regulated by EGR1 is differentially regulated in miR-146a-deficient tumors

**Figure 5:**
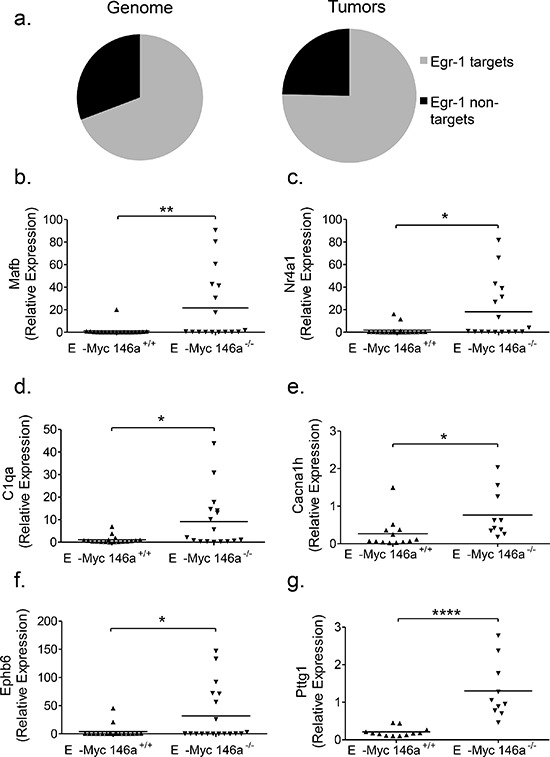
Genes with EGR1 transcription factor binding sites are enriched in Eμ-Myc driven tumors with miR-146a deficiency **a.** Graphical representation of the percentage of the genome (left) containing EGR1 TFBS (11136 TFBS out of 16288 genes that were conserved between human and mouse), compared with the percentage of differentially regulated genes in the tumor dataset (right) that show EGR1 TFBS (153 with TFBS out of 203 differentially regulated genes that are conserved between mouse and human). This difference was found to be statistically significant (Chi-square test, one-sided *p* = 0.0353). **b–g.** RT-qPCR confirmation of differentially regulated genes that show EGR1 TFBS, including *Mafb* (b; *t*-test, *p* = 0.0093), *Nr4a1* (c, *t*-test, *p* = 0.0126), *C1qa* (d; *t*-test, *p* = .0101), *Cacna1 h* (e; *t*-test, *p* =.0308), *Ephb6* (f; *t*-test, *p* = .0248), *Pttg1* (g; *t*-test, *p* < 0.0001). For these RT-qPCR analyses, *n* = 18 for Eμ-Myc miR-146a^+/+^ and *n* = 17 for Eμ-Myc miR-146a^−/−^.

The early growth response-1 gene (*Egr1)*, has previously described functions in hematopoietic differentiation [25, 26]. Given that *Egr1* is overexpressed in miR-146a-deficient tumors, we undertook an analysis to determine whether the *Egr1* transcriptome is differentially regulated. Using a publically available ChIP-Seq dataset, we gathered a list of EGR1 transcription factor binding sites (TFBS) around known protein coding genes in three different human cell lines (K562, GM12878, and H1-hESC) [27]. This dataset was compared to the list of differentially regulated genes in miR-146a-deficient tumors that have human homologs (Supplementary Figure 6a–6b). Remarkably, genes that show TFBS for EGR1 were statistically overrepresented in the differentially regulated gene set from miR-146a-deficient tumors (Figure 5a). We then confirmed several targets of EGR1 that (i) were differentially regulated in the RNA-sequencing dataset and (ii)had been previously shown in the literature to be EGR1 targets or had EGR1 binding sites based on the ChIP-Seq datasets. Several genes that are important in hematopoiesis and/or cancer were profiled in the larger set of tumors and showed a significantly differential regulation. These genes included *Mafb* (Figure 5b), *Nr4a1* (Figure 5c), *C1qa* (Figure 5d), *Cacna1 h* (Figure 5e), *Ephb6* (Figure 5f) and *Pttg1* (Figure 5g and Supplementary Figure 6c). Changes in gene expression in Egr1 and a subset of its targets were conserved in the subset of tumors derived from female mice, hinting that these molecular changes may underlie the increased lethality in the knockout mice (Supplementary Figure 5k–5n). Together, these findings indicate that miR-146a-regulated *Egr1* may represent a critical target that leads to the elaboration of a gene expression signature and the more aggressive phenotype observed during miR-146a-deficient, Eμ-Myc-mediated oncogenesis.

### *Egr1* is regulated by miR-146a and overexpression of miR-146a has an anti-growth effect on B-cell lymphoma cell lines

**Figure 6:**
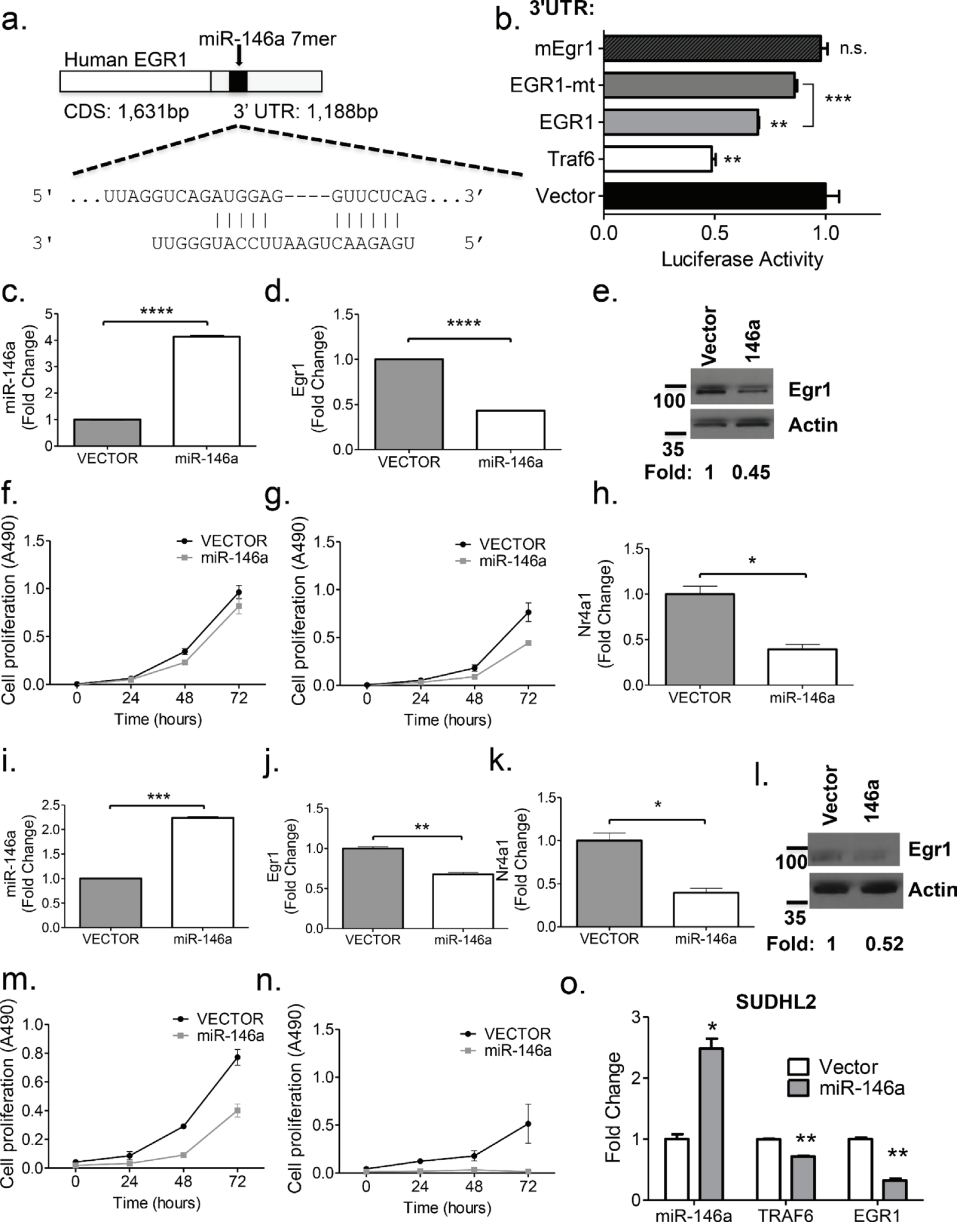
Egr1 is regulated by miR-146a **a.** The human EGR1 cDNA contains a 1, 188 bp 3′UTR that contains an intact miR-146a 7-mer binding site. Shown is a schematic of the binding between EGR1 and miR-146a. **b.** Luciferase assays quantitating repression with MGP/miR-146a co-transfection relative to MGP alone for each of the UTRs depicted. Each measurement is representative of firefly luciferase normalized to renilla luciferase, and was performed in duplicate, with the experiment was repeated at least three times (*T*-test; Traf6 v Vector, *p* = 0.0013; EGR1 vs. vector, *p* = 0.0077; EGR1 vs. mutant EGR1, *p* = 0.0002). **c–d.** RT-qPCR analyses of miR-146a and *Egr1*, respectively in the murine 70Z/3 cell line. **e.** Western Blot analysis confirms EGR1 repression with miR-146a over expression in the 70Z/3 cell line. Fold repression was computed using ImageJ software. **f–g.**. Cell proliferation (MTS) assays were performed using 70Z/3 cells transduced with either empty vector (MGP) or miR-146a over expressing vector (MGP-miR-146a). Basal growth is shown in (f), while growth in samples following 24 hours of serum starvation is shown in (g). **h.** RT-qPCR of the EGR1 target gene, *Nr4a1*, shows repression in miR-146a overexpressing cells. **i–k.** RT-qPCR analyses of miR-146a, *Egr1* and *Nr4a1*, respectively, in the murine WEHI-231 cell line. **l.** Western Blot analysis, as in (e), using the WEHI-231 cell line **m–n.** Cell proliferation (MTS) assays were used to measure basal growth and growth following 4 hours of serum starvation **o.** miR-146a overexpression in a human DLBCL cell line, SUDHL2 results in the repression of TRAF6 and EGR1. All comparisons were made with *T*-test for Figure 6c-o, with the following legend: *p < 0.05; **p < 0.005; ***p < 0.0005; ****p < 0.0001.

To elucidate whether miR-146a targets *Egr1*, we examined the 3′ untranslated region (UTR) of the cDNA transcript. In the human *EGR1* sequence, there is a miR-146a 7-mer binding site located at position 111–117 of the 3′ UTR (Figure 6a). The DNA sequence surrounding this area is somewhat conserved between the human and the mouse, but the complete 7-mer site is not present in the mouse (Supplementary Figure 6d). To examine direct targeting, we cloned a 996 bp segment of the human *EGR1* 3′UTR into the pmiRGlo vector. Co-transfection of a miR-146a over- expression vector along with the luciferase-*EGR1* 3′UTR fusion construct showed significant repression of luciferase activity, compared to the empty vector, similar to that observed for *Traf6*. Mutation of the binding site for miR-146a in the EGR1 3′ UTR derepressed luciferase expression. A similar repression was not consistently observed for the murine Egr1 3′UTR (Figure 6b). Stable overexpression of miR-146a using a retroviral vector in the murine leukemia cell lines, 70Z/3 and WEHI-231 led to a repression of *Egr1* at both the transcript and protein levels (Figure 6c–6e, 6i–6j, 6l). In addition, overexpression of miR-146a led to decreased growth of both cell lines at baseline and following serum starvation (Figure 6f–6g, 6m–6n). Moreover, we observed repression of the EGR1 target, *Nr4a1* (Figure 6h, 6k), implicating the same sequence of regulation with miR-146a overexpression as that observed with miR-146a deficiency in the tumors. Moreover, miR-146a overexpressing cells showed a downregulation of Blimp1 and Bcl6 (Supplementary Figure 6i–6l), in line with the observations made in the tumors. In the human DLBCL cell line, SUDHL2, miR-146a overexpression led to repression of EGR1 as well as the expected target of miR-146a, TRAF6 (Figure 6o). Nrp2 was validated as an additional target of miR-146a (Supplementary Figure 6e–6h). These findings imply a role for miR-146a in the regulation of B-cell leukemia/lymphoma cell growth and demonstrate that in the human, miR-146a directly targets EGR1 via canonical 3′ UTR-mediated targeting. Hence, miR-146a overexpression and knockout results in significant effects on Egr1 and downstream gene expression, suggesting a conserved regulatory module in the human and mouse.

## DISCUSSION

In this manuscript we describe the modulation of tumorigenesis by the NF-κB induced tumor suppressor microRNA, miR-146a. miR-146a plays a very important role in immune cells and seems to be critical in modulating feedback inhibition of the NF-κB pathway. Its role in T-cells, myeloid cells and hematopoietic stem cells is well-established, with deletion of this miRNA leading to T-cell hyper activation, myeloid hyperplasia and tumors, and stem cell exhaustion [9, 11, 14, 16]. The role of miR-146a in the developmental sequence of B-cells is less understood. In young miR-146a-deficient mice, B-cell development appears to proceed normally, but by the age of six months, lymphoid follicles in the spleen and other lymphoid tissue demonstrate hyperplasia [9, 28]. Following this phase, myeloproliferative disease becomes the dominant phenotype and B-cell numbers drop as the mice age. Nonetheless, aged miR-146a-deficient mice show an increased incidence of B-cell malignancies. Interestingly, these tumors show a predilection for the lymph nodes, similar to what we have observed here with Eμ-Myc driven tumors.

miR-146a-deficient Eμ-Myc transgenic mice develop mature B-cell neoplasms with IgM and/or CD138 expression, leading to a higher proportion of lymph node tumors and leukemia in the peripheral blood. There is some heterogeneity in the proportion of Eμ-Myc mice reported to develop IgM+ tumors in the literature [29, 30], but our results have been consistently in the 20–30% range. The immunophenotypic differences, along with concordant gene expression changes (e.g., Blimp1 and Bcl6), indicate that miR-146a deficiency may alter the stage of B-cell development that is most susceptible to transformation by *c-Myc*. This is an interesting observation as B-cell neoplasms in humans that have increased levels of *c-Myc* can also derive from different stages of development (for example, B-lymphoblastic leukemia, Burkitt's lymphoma, DLBCL, and plasma cell myeloma) [31–35]. Here, the expression of a miRNA in an experimental model of Myc-mediated oncogenesis does alter the stage of B-cell oncogenesis. Although the relevance to human disease remains to be established, it is interesting to speculate that miRNA expression may have an important role in defining the cellular composition of lymphoma from a given driver mutation.

An important question raised by this study is whether the observed phenotypes occur as a consequence of cell-intrinsic or cell-extrinsic mechanisms. The primary tumor sites showed primarily B-cells, and histologically, the tumors appeared to be quite homogeneous. In miR-146a-deficient mice, T-cell activation is thought to occur as a consequence of repeated bouts of subclinical infection and inflammation without the “recalibrating” effects of miR-146a expression. We do not think this is a likely cause for the augmentation of oncogenesis, since the development of tumors under specific pathogen free conditions do not occur early in life in miR-146a singly-deficient mice. Heterozygotes did not show increased mortality in the presence of Eμ-Myc, whereas miR-146a heterozygosity alone causes inflammatory changes [9]. Hence, it is likely that increased tumorigenesis in our mice occurs primarily as a consequence of B-cell intrinsic mechanisms. However, we cannot entirely exclude a cell-extrinsic process driven by benign but hyperactivated T-cells. Future studies to address this issue will include producing B-cell specific knockouts and knock-ins of miR-146a to study disease progression, but are beyond the scope of the current study.

In an effort to further characterize tumorigenesis in these mice, we undertook gene expression analysis by high-throughput sequencing. We have found that a small set of genes are significantly differentially regulated between miR-146a sufficient and deficient Eμ-Myc tumors. While the functional analysis did not reveal an overall pattern to the differentially regulated gene set, the individual genes do seem to be important in various aspects of tumorigenesis (Supplementary Table 2) [36, 37]. Amongst the differentially regulated genes, many have roles in oncogenesis and B-cell development. Perhaps the most interesting gene to be identified by our analysis is *Egr1*, a factor known to promote differentiation in the hematopoietic lineage. EGR1 transcriptionally induces a range of genes, and the differentially regulated gene set in miR-146a-deficient mice was enriched for these targets. Indeed, some of the most differentially regulated genes in our dataset were previously described targets of EGR1 or putative targets as defined by the presence of transcription factor binding sites. Critically, miR-146a regulates *Egr1*, and provides an explanation for the observed phenotypic differences in the tumors from these mice. However, we must note that direct targeting was only seen with the human EGR1 3′UTR, and hence the mechanism of this regulation in the mouse may be indirect. This could include non-canonical mechanisms of miRNA targeting (such as in the 5′UTR) and/or indirect regulation.

miR-146a overexpression changes the growth of murine B-cell lines, suggesting the importance of the EGR1-mediated transcription program in maintaining growth of these cells. Notably, miR-146a overexpression also led to the repression of certain mRNAs that are important in B-cell differentiation including Blimp1 and Bcl6, once again supporting the notion of miR-146a playing a role in the maturation stage of the tumor cells. This is line with prior reports showing that expression of an Egr1 transgene supported the development of progenitor cells into mature, IgM-expressing B-cells [26]. Interestingly, some of the genes that contain TFBS for EGR1 are also regulated by miR-146a (for example, *Nrp2*), suggesting that miR-146a may target several points in the same pathway during B-cell oncogenesis. Downstream, genes without a defined role in B-cell neoplasms were also identified. *Pttg1* is overexpressed in a wide variety of endocrine and non-endocrine tumors, modulates tumor invasiveness and recurrence in several systems, and has functions in chromatid separation and cell cycle progression [38]. *Jhy* is another gene we identified whose deficiency causes juvenile hydrocephalus in mice [39]. It will be of great interest to study how miR-146a deficiency causes differential regulation of these novel genes and what their roles are in normal and malignant B-lymphopoiesis.

Our findings also point to the cell-type specific nature of miRNA mediated regulation. The targets uncovered in a malignant B-cell are different than those found in an activated T-cell or a myeloid cell. For example, our findings suggest that *Traf6* and *Irak1*, which are highly important in the elaboration of myeloid phenotypes, may not be as important in B-cell oncogenesis, particularly that induced by *c-Myc*, as these genes were not differentially regulated in the tumors that we examined (data not shown). These findings highlight the need for experimental work in carefully defined physiological and pathological systems to comprehensively understand miRNA function.

In summary, we show that concurrent *c-Myc* overexpression coupled with the absence of a *bona fide* tumor suppressor miRNA leads to more aggressive tumor due to a small set of genes that are regulated directly or indirectly by miR-146a. Our novel set of targets may indicate that miR-146a regulates components of signaling networks other than the NF-κB inflammatory pathway. Hence, our work opens the door to new areas of investigation in B-cell oncogenesis and miRNA biology.

## MATERIALS AND METHODS

### Mice

miR-146a-deficient (miR-146a^−/−^) mice were developed as previously described [9, 11, 16]. Eμ-Myc mice were purchased from Jackson laboratories and housed under pathogen free conditions at the University of California, Los Angeles [40]. Eμ-Myc and miR-146a^−/−^mice were bred to obtain Eμ-Myc miR-146a^+/−^mice with further miR-146a^−/−^ intercross producing Eμ-Myc miR-146a^−/−^ mice. Mice were monitored for tumors and sacrificed when they became pre-moribund indicated by the following criteria: tumors larger than 1.5cm, emaciation, or any other signs of distress. All mouse studies were approved by the UCLA Office of Animal Research Oversight.

### Flow cytometry

Blood, bone marrow, spleen, and lymph node tumors were collected from the mice under sterile conditions. Single cell suspensions were lysed in red blood cell lysis buffer. Fluorochrome conjugated antibodies against B220, CD3ε, CD11b, Ter119, CD19, IgM, CD80, CD138, CD44, CD21, CD23, and CD5 were used for staining (all antibodies obtained from Biolegend). Flow cytometry was performed on a FACSAria and analysis performed using FlowJo software. Dichotomization of flow cytometric measurements was accomplished by visual inspection of the data and identification of clusters within the data. These were then validated by comparison of the means and averages of the two clusters. For CD138, this was accomplished by examining the Mean Fluorescence Intensity and determining that the low expression cluster had a mean MFI, 154.0 ± 13.96 (N = 30) and 557.6 ± 71.87 (*N* = 7) for the high CD138 samples (*p* < 0.0001 for this comparison).

### Histopathology

Organs were collected after necropsy and fixed in 10% neutral buffered formalin. These were then embedded in paraffin, processed for hematoxylin and eosin staining by the Translational Pathology Core Laboratory at UCLA. Histopathologic analysis was performed by a board certified hematopathologist (D.S.R). The degree of splenic involvement was scored on a 4-point scale for red and white pulp involvement. Analysis of dichotomized or ordinal-type histopathologic data was accomplished by the use of Fisher's Exact Test.

### Statistical analyses

Figures are graphed as mean with the standard deviation of the mean (SD) for continuous numerical data. Bar graphs are employed to show dichotomized or ordinal-type histopathologic data. Student's *t*-test, Fisher's exact test, Chi square test, and Kaplan-Meier survival analysis were performed using GraphPad Prism software, applied to each experiment as described in the figure legends.

### RNA-sequencing and analysis

Total RNA was extracted from tumors using Trizol combined with Qiagen miRNEasy mini kit with additional on column DNAse I digestion. Following isolation of RNA, cDNA libraries were built using the Illumina(San Diego, CA) TrueSeq RNA Sample Preparation kit V2 (RS-122–2001). An Agilent Bioanalyzer was used to determine RNA quality (RIN > 8) prior to sequencing. RNA-Seq libraries were sequenced on an Illumina HiSeq 2000 (single-end 100bp). Raw sequence files were obtained using Illumina's proprietary software and will be made available at NCBI's Gene Expression Omnibus resource. RNA-Seq reads were aligned using STAR v2.3.0 [41]. The GRCm38 assembly (mm10) of the mouse genome and the junction database from Ensembl's gene annotation (release 71) were used as reference for STAR. The count matrix for genes in Ensembl's genome annotation (excluding rRNAs, Mt_rRNAs and Mt_tRNAs) was generated with HTSeq-count v0.5.4p3 (http://www-huber.embl.de/users/anders/HTSeq/) and normalized using the geometric mean across samples [42]. DESeq v1.14.0 [42] was used to classify genes as differentially expressed (Benjamini-Hochberg adjusted *p*-value < 0.05). Moderate fold changes between conditions were obtained from variance-stabilized data [42]. Functional annotation of differentially expressed genes was generated through the use of DAVID [36, 37]. Hierarchical gene clustering was performed with GENE-E (http://www.broadinstitute.org/cancer/software/GENE-E/). To display the heatmap, the expression levels were re-scaled so that, for each gene, the limits of the color scale correspond to the minimum and maximum expression levels across all samples.

### EGR1 transcription factor binding site analysis

Publically available ENCODE data for EGR1 Transcription Factor Binding Site ChIP-Seq Uniform Peak analysis was downloaded from the UCSC Genome Browser for the K562, H1-hESC, and GM12878 cell lines [27]. For each line, the locations for all EGR1 Transcription Factor Binding Sites (TFBS) were grouped based on the closest known gene based using the UCSC Main (hg19) ccds gene list. The genes with one or more TFBS were compared to the mouse (mm10) RNASeq data set to identify genes that were differentially expressed in the miR-146a-deficient tumors and also had at least one EGR1 TFBS in close proximity to the gene (defined as 3 kb). A Chi-Square test was performed with one degree of freedom to compare the relative frequency of EGR1 TFBS in the differentially expressed dataset (Observed) with the frequency across the genome (Expected). Only the mouse genes with a human homolog (total of 16288 genes) were used.

### RT-qPCR

RNA collected from the murine tumors was reverse transcribed using qScript reagent and PerfeCTa SYBR Green FastMix reagent (Quanta Biosciences) or TaqMan MicroRNA Assay (Life Technologies). Primer sequences used are listed in Supplementary Table 1.

### Western blot

Tumor cell suspensions were lysed in RIPA buffer (Boston BioProducts) supplemented with Halt Protease and Phosphatase Inhibitor Cocktail (Thermo Scientific). Equal amounts of protein lysate (as quantified by using bicinchoninic acid protein assay, BCA (Thermo Scientific)) were electrophoresed on a 5–12% SDS–PAGE and electroblotted onto a nitrocellulose membrane. Antibodies used were c-MYC Rabbit polyclonal (#9402), EGR1 (44D5) Rabbit monoclonal (all antibodies from Cell Signaling), and ß-Actin (AC15) mouse monoclonal antibody (Sigma Aldrich). Secondary HRP-conjugated antibodies were purchased from Santa Cruz Biotechnology.

### MTS assay

Cell proliferation was measured using the Promega Cell Titer 96 Aqueous Non-Radioactive Cell Proliferation Assay kit. After addition of reagent according to the manufacturer's protocol, cells were incubated at 37°, 5% CO_2_ for 4 hours and absorbance was measured at 490 nm.

### Luciferase assays

A 996-bp segment of the human *EGR1* 3′UTR containing the miR-146a site was cloned into the pmiRGlo dual luciferase vector (Promega). A similar cloning strategy was used to clone murine Egr1 3′UTR and the Nrp2 UTR (see Supplementary Table 1). For mutation of the miR-146a binding site, we utilized site-directed mutagenesis as previously described using the primers shown in Supplementary Table 1 [43]. Co-transfections were performed with Lipofectamine 2000 (Life Technologies) as per the manufacturer's instructions. Cells were lysed after 24 hours, substrate was added and luminescence was measured on a Glomax-Multi Jr (Promega).

### Genotyping for miR-146a mice and c-Myc mice

Mice were genotyped for miR-146a deletion and Eμ-Myc presence using DNA extracted from tail samples. Genotyping for miR-146a deletion was done as described previously [11]. Primers are listed in Supplementary Table 1.

## SUPPLEMENTARY FIGURES AND TABLES


