# Supplementary material for: MicroRNA-146a modulates B-cell oncogenesis by regulating Egr1
Source: Oncotarget. 2015 Apr 13;6(13):11023–37. doi: 10.18632/oncotarget.3433 (PMC4484436; doi:10.18632/oncotarget.3433)
Supplement: Supplementary file 1 [file oncotarget-06-11023-s001.pdf]

SUPPLEMENTARY FIGURES AND TABLES

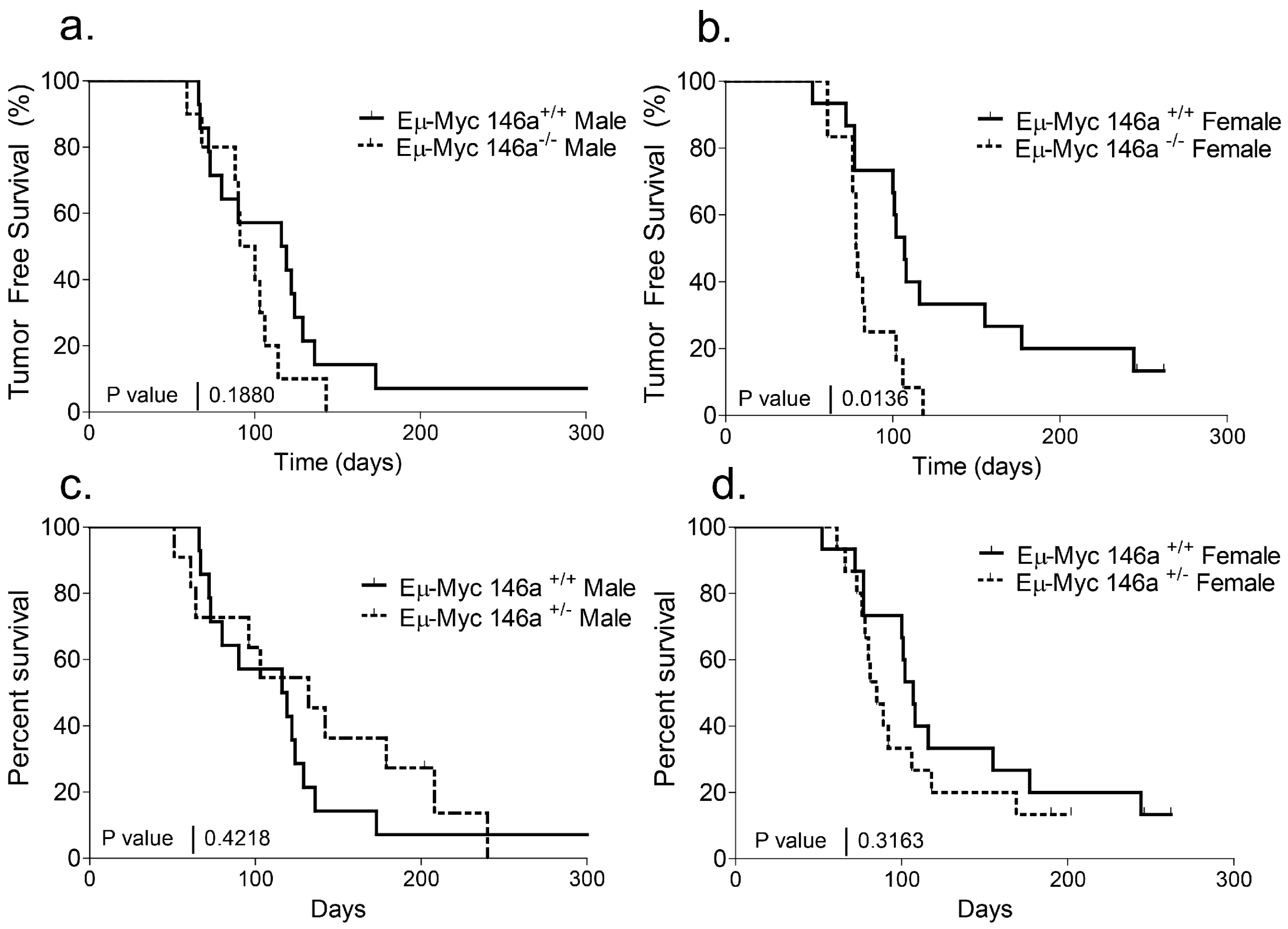

Supplementary Figure S1: miR-146a deficiency causes increased mortality in female Eμ-Myc mice. (a) Tumor-free survival curve of male mice with Eμ-Myc oncogene and either wild-type or homozygous deficiency of miR-146a (n = 14 for Eμ-Myc miR-146a$^{+/+}$ n = 10 for Eμ-Myc miR-146a$^{-/-}$: Log-Rank Test, p = 0.1880). **(b)** Tumor-free survival curve of female mice with Eμ-Myc oncogene and either wild-type or homozygous deficiency of miR-146a (n = 15 for Eμ-Myc miR-146a$^{+/+}$, n = 12 for Eμ-Myc miR-146a$^{-/-}$; Log-rank Test, p = 0.0136). **(c)** Tumor-free survival curve of male mice with Eμ-Myc oncogene and either wild-type or heterozygous deficiency of miR-146a (n = 14 for Eμ-Myc miR-146a$^{+/+}$, n = 11 for Eμ-Myc miR-146a$^{+/-}$; Log-Rank Test, p = 0.4218). **(d)** Tumor free survival curve of female mice with Eμ-Myc oncogene and either wild-type or heterozygous deficiency of miR-146a (n = 15 for Eμ-Myc miR-146a$^{+/+}$, n = 15 for Eμ-Myc miR-146a$^{+/-}$; Log-Rank Test, p = 0.3163).

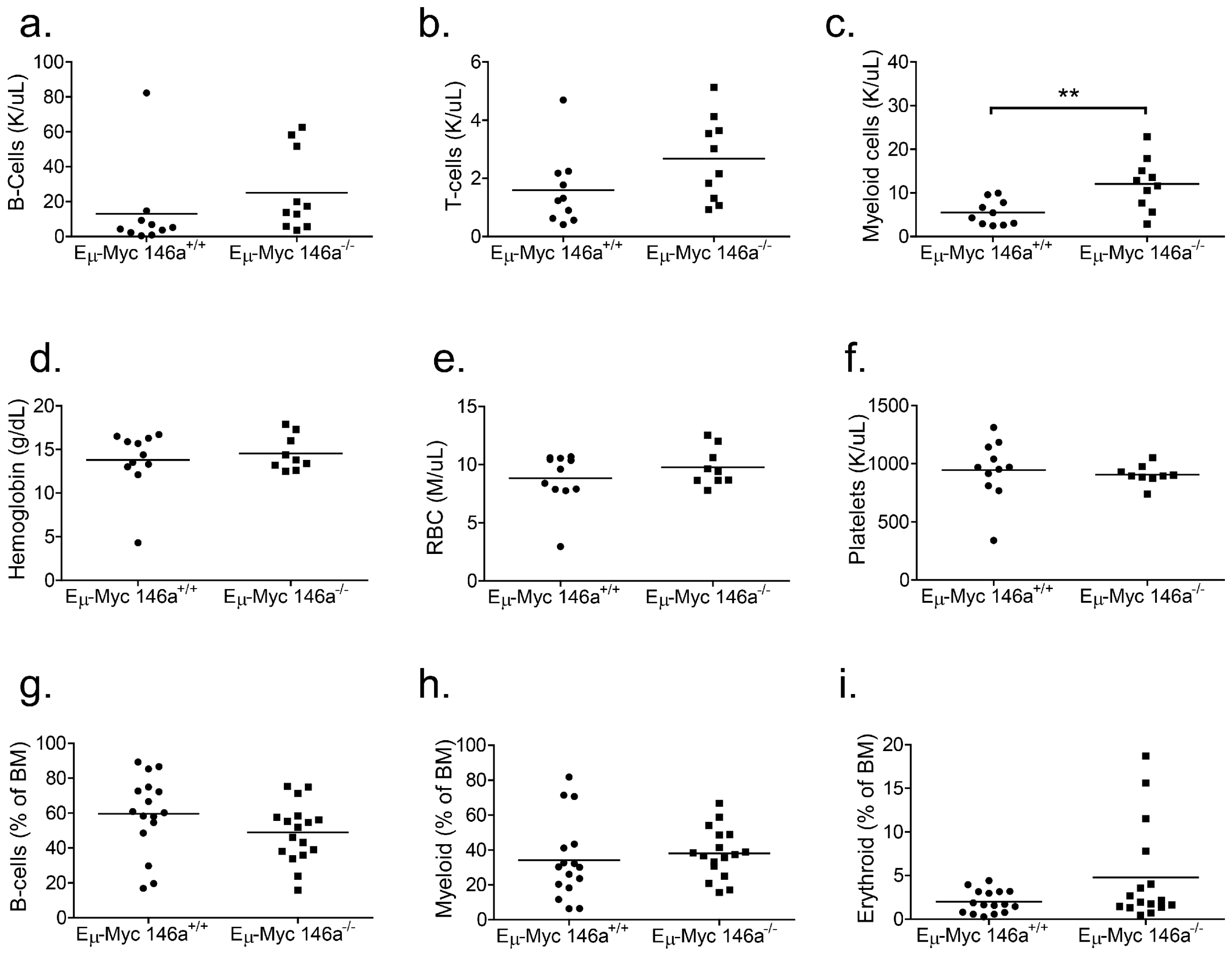

Supplementary Figure S2: Blood and bone marrow composition in Eμ-Myc animals at the time of death. (a–c) Quantitation of absolute number of B-cells (a), T-cells (b), and Myeloid cells (c) based on CBC and FACS analysis of blood at time of death (n = 10 Eμ-MycmiR-146a$^{+/+}$, and n = 10 Eμ-Myc miR-146a$^{-/-}$; t-test p = 0.2719, 0.0904 and 0.0052 respectively). **(d–i)** Hemoglobin levels, red blood cell, and platelet counts at time of death (n = 11 Eμ-MycmiR-146a$^{+/+}$, and n = 9 Eμ-Myc miR-146a$^{-/-}$; t-test, p = 0.5663, 0.3150 and 0.6521 respectively). Percentage of B-cells (g), Myeloid (h), and Erythroid (i) cells in the bone marrow of mice at time of death (n = 16 Eμ-Myc miR-146a$^{+/+}$, and n = 17 Eμ-Myc miR-146a$^{-/-}$; t-test, p = 0.1250, 0.5529 and 0.0635 respectively).

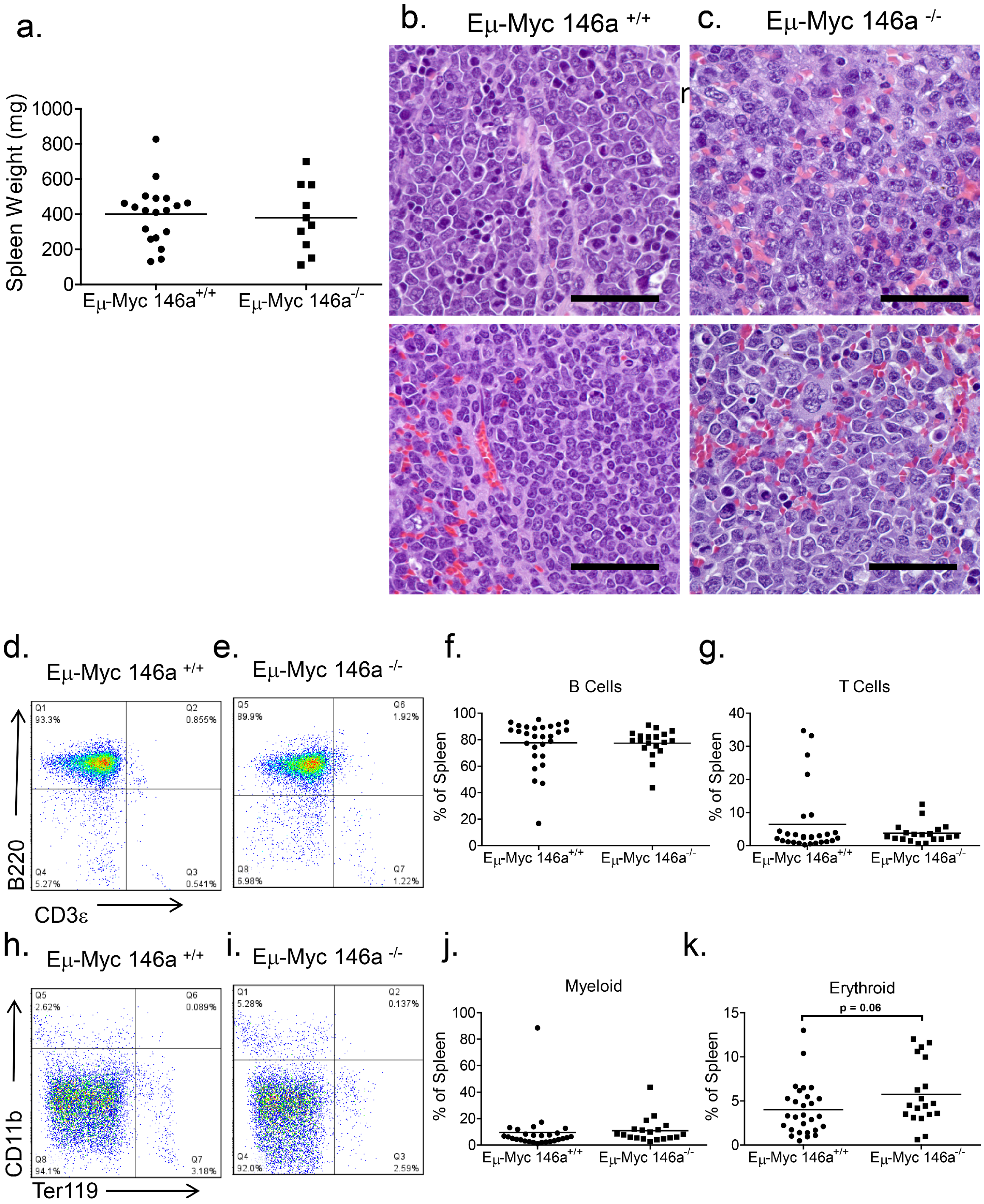

Supplementary Figure S3: Analysis of spleens from Eμ-Myc animals deficient for miR-146a. (a) Spleen weights at the time of death for all animals where data was available (n = 19 Eμ-Myc miR-146a$^{+/+}$ and n = 10 Eμ-Myc 146a$^{-/-}$; t-test, p = 0.7662). **(b–c)** Representative high power images of paraffin embedded H&E stained tumor samples showing red (top panels), and white (bottom panels) pulp of splenic tumors from Eμ-Myc animals sufficient or deficient for miR-146a. **(d–e, h–i)** Representative FACS plots from Eμ-Myc miR-146a$^{+/+}$ and Eμ-MycmiR-146a$^{-/-}$ spleens stained with B220 and CD3e, or CD11b and Ter119, respectively. **(f–g, j–k)** Quantitation of the percentage of B-lymphocytes, T-lymphocytes, Myeloid or Erythroid cells in the spleens of Eμ-Myc miR-146a$^{+/+}$ and Eμ-MycmiR-146a$^{-/-}$ mice, based on FACS (n = 28, Eμ-Myc miR-146a$^{+/+}$, and n = 19, Eμ-Myc miR-146a$^{-/-}$; t-test, p = 0.9505, 0.2568, 0.7114, and 0.0600 respectively).

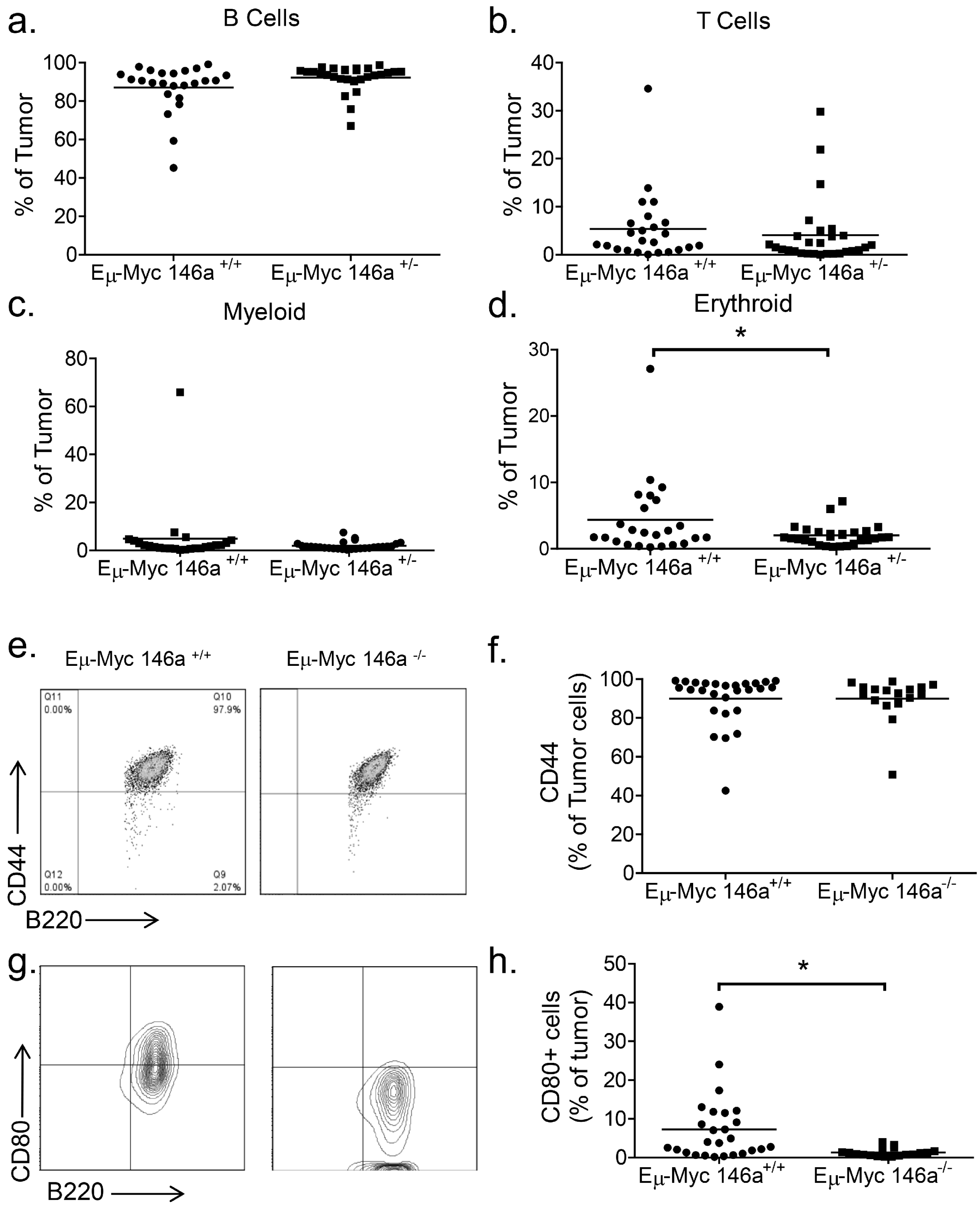

Supplementary Figure S4: Immunophenotypic properties of tumors in mice with heterozygous and homozygous deficiency of miR-146a. (a–d) B-, T-, myeloid, and erythroid cells, respectively, quantitated by FACS in tumors from mice carrying the Eμ-Myc oncogene and wild-type or heterozygous for miR-146a ($n = 24$ for Eμ-Myc miR-146a$^{+/+}$ and $n = 28$ for Eμ-Myc miR-146a$^{+/–}$; *t*-test $p = 0.0704$, 0.5164 and 0.2368, 0.0427 respectively). **(e)** Representative FACS plots from Eμ-Myc 146a$^{+/+}$ or Eμ-Myc 146a$^{–/–}$ tumors stained with CD44 and B220. **(f)** Quantitation of all mice where data was available for the percent of CD44 positive cells in tumors from Eμ-Myc 146a$^{+/+}$ or Eμ-Myc 146a$^{–/–}$ ($n = 27$ Eμ-Myc 146a$^{+/+}$ and $n = 17$ Eμ-Myc 146a$^{–/–}$; *T*-Test, $p = 0.9922$). **(g)** Representative FACS plots from Eμ-Myc 146a$^{+/+}$ or Eμ-Myc 146a$^{–/–}$ tumors stained with CD80 and B220. **(h)** Quantitation of all mice where data was available for the percent of CD44 positive cells in tumors from Eμ-Myc 146a$^{+/+}$ or Eμ-Myc 146a$^{–/–}$ ($n = 27$ Eμ-Myc 146a$^{+/+}$ and $n = 17$ Eμ-Myc 146a$^{–/–}$; *T*-Test, $p = 0.0069$).

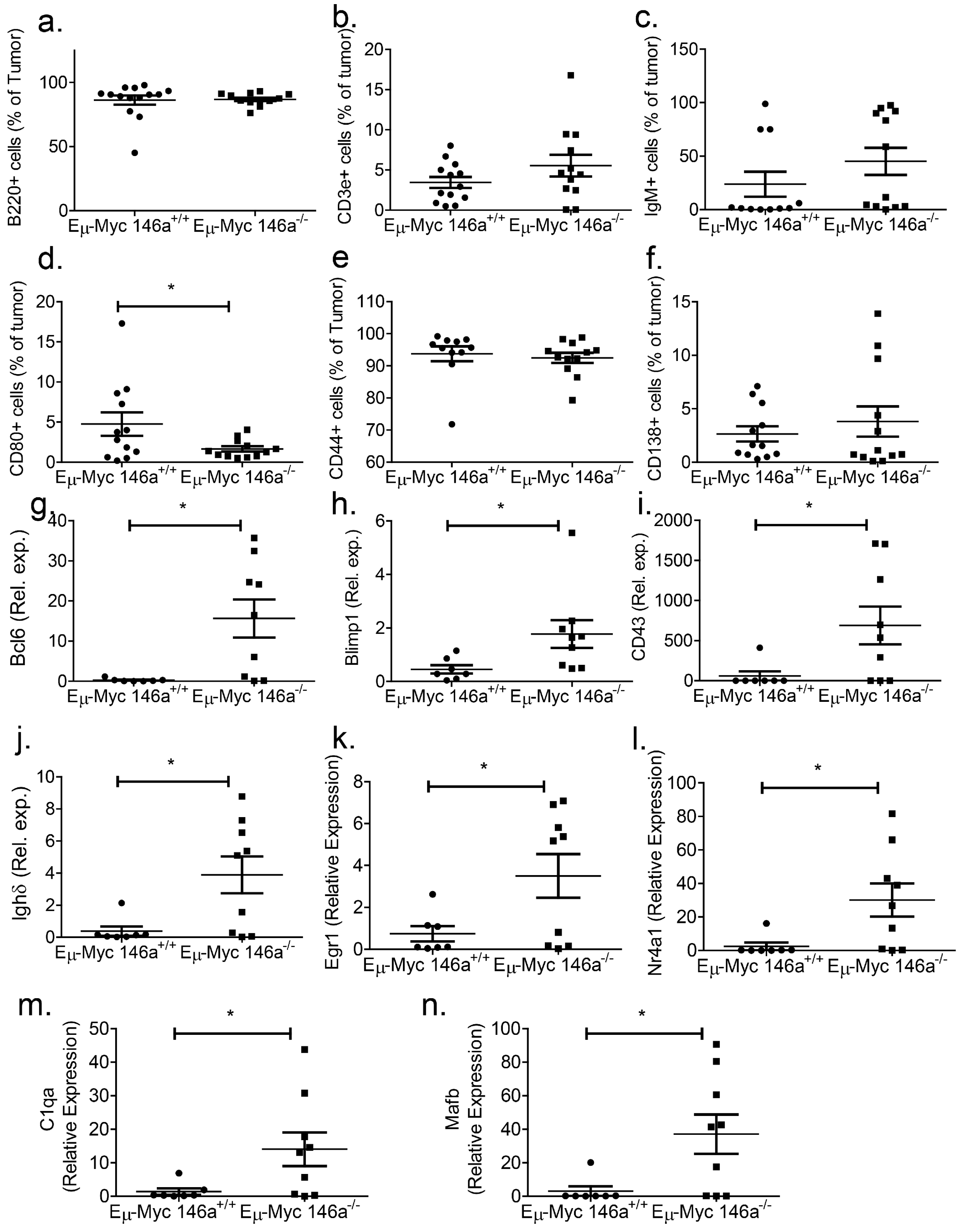

Supplementary Figure S5: Analysis of immunophenotypic and gene expression features of tumors from female Eμ-Myc 146a$^{-/-}$ mice. (a–f) Quantitation of B220+ (a), CD3e+ (b), IgM+ (c), CD80+ (d), CD44+ (e) and CD138+ (f) cells from female mice that carry the Eμ-Myc transgene and are either sufficient or deficient for miR-146a. Only CD80 expression is significantly different (*T*-test, $p = 0.0487$). This may be due to the reduced numbers of animals available for the analysis ($n = 12$ Eμ-Myc 146a$^{+/+}$ and $n = 12$ Eμ-Myc 146a$^{-/-}$). **(g–j)** RT-qPCR analyses of B-cell maturation associated transcripts from female mice that carry the Eμ-Myc transgene and are either sufficient or deficient for miR-146a. All comparisons showed a statistically significant difference ($p < 0.05$ for all comparisons between the mice; $n = 7$ Eμ-Myc 146a$^{+/+}$ and $n = 9$ Eμ-Myc 146a$^{-/-}$). **(k–n)**. RT-qPCR analyses of Egr1 and putative Egr1-regulated transcripts from female mice that carry the Eμ-Myc transgene and are either sufficient or deficient for miR-146a. All comparisons showed a statistically significant difference ($p < 0.05$ for all comparisons between the mice; $n = 7$ Eμ-Myc 146a$^{+/+}$ and $n = 9$ Eμ-Myc 146a$^{-/-}$).

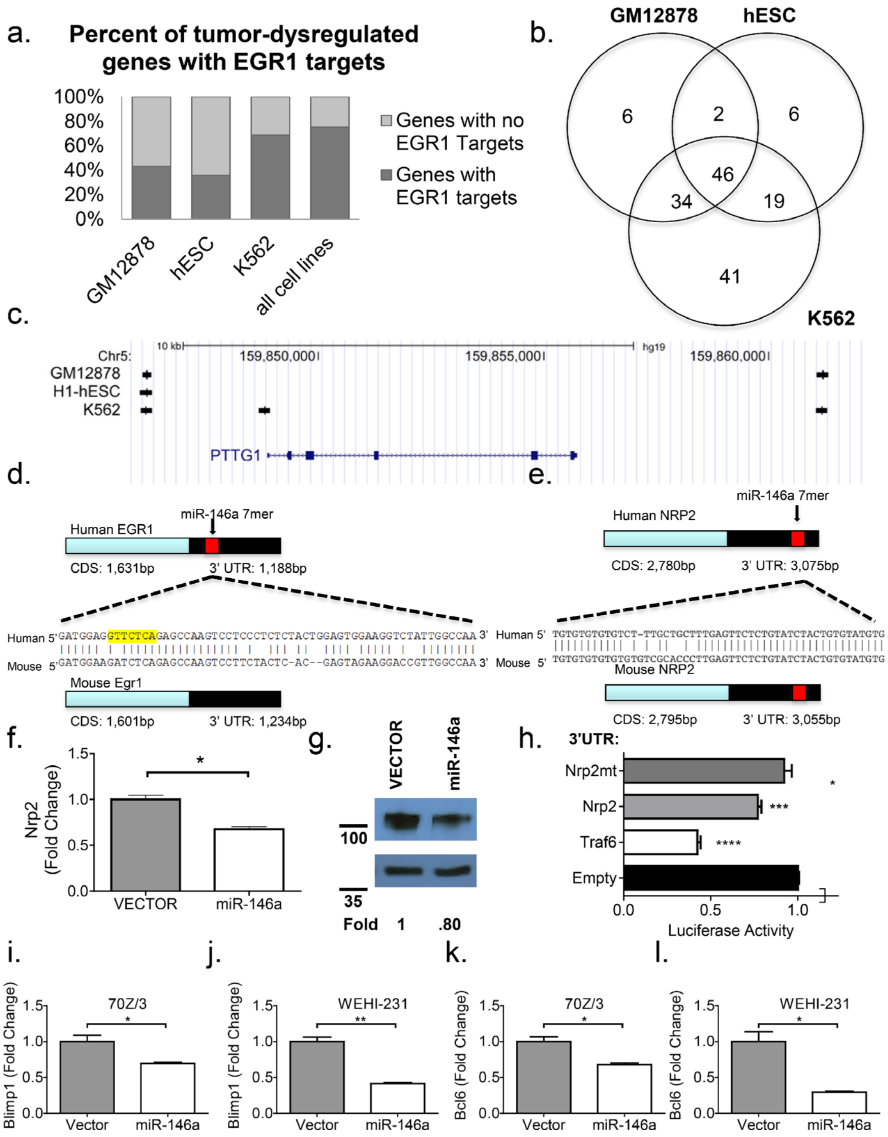

Supplementary Figure S6: High throughput analyses for EGR1 TFBS and demonstration of a novel miR-146a target, *Nrp2*. **(a)** Plot showing the numbers of genes that were differentially regulated in miR-146a deficient tumors that have EGR1 binding sites in the three different cell lines. Note that the K562 cell line had the highest number of binding sites. The cell lines displayed a wide range in the total number of EGR1 transcription factor binding sites (TFBS). The K562 cell line had a total of 36,367 sites representing 12,741 genes, the GM12878 line had 16,530 sites representing 9,170 genes, and the H1-hESC line had only 8, 818 sites representing 6,349 genes. There exists a wide range in the total number of EGR1 TFBS sites as well as the total genes with EGR1 binding sites. In the K562 cell line, 62% of total protein coding genes are putatively regulated by EGR1. In the H1-hESC line 31% of genes have EGR1 TFBS and in GM12878 line 45% of genes have EGR1 TFBS sites. When all three cell lines are combined, there are a total of 62,071 sites representing 13,944 unique genes. **(b)** Intersection of differentially regulated genes from the RNA-Seq dataset overlaid with the genes containing EGR1 TFBS obtained from ChIP-Seq data of the three different cell lines. **(c)** *Pttg1* is a differentially regulated gene in miR-146a deficient tumors. It is found on chromosome 5 and has EGR1 transcription factor binding sites based on the reanalyzed ChIP-Seq data presented above. **(d)** Schematic representation of human and mouse *Egr1* gene showing the miR-146a binding site in the 3′UTR. **(e)** Schematic representation of human and mouse *Nrp2* gene showing the miR-146a binding site in the 3′UTR. **(f)** RT-qPCR analysis of *Nrp2* in 70Z/3 cell lines either expressing MGP or MGP-miR-146a vector (*t*-test, $p = 0.0148$). **(g)** Western blot analysis for NRP2 after miR-146a shows reduction in the protein levels in 70Z/3 cells when compared to the reference gene actin (upper panel: Nrp2 and lower panel, β-actin). Shown below are fold repression computed using ImageJ software. **(h)** Luciferase assays showing repression seen with MGP/miR-146a co-transfection relative to MGP alone for each of the UTRs depicted. Each measurement is representative of firefly luciferase normalized to renilla luciferase, and was performed in duplicate, with the experiment was repeated at least three times (*T*-test; Traf6 v Vector, $p < 0.0001$; Nrp2 vs. vector, $p = 0.0005$; Nrp2 vs. mutant Nrp2, $p = 0.033$). **(i–l)**. RT-qPCR analyses of Blimp1 and Bcl6 in 70Z/3 and WEHI-231 cells that are overexpressing miR-146a. All comparisons showed statistically significant downregulation of these genes in miR-146a overexpressing cell lines (*$p < 0.05$; **$p < 0.005$).

Supplementary Table S1: RT-qPCR primers and genotyping primers used. Listed are the primers used for RNAseq data validation, genotyping miR-146a and Eμ-Myc allele, and cloning. 5'*P** indicates phosphorylated 5' end.

RT-qPCR primers	Direction	Sequence
Jhy	FOW	5′ GGTGCCGGCAGGATGAATAA 3′
	REV	5′ AAGTTGGTGTGATGGACGGG 3′
Cacna1h	FOW	5′ ATGCTTGGGAACGTGCTTCTT 3′
	REV	5′ GTCTGGTAGTATGGCCGCAA 3′
Camk2b	FOW	5′ TGGTGGAACAAGCCAAGAGTTT 3′
	REV	5′ GAGGGAGAGATCCTTTGGGG 3′
Myo18b	FOW	5′ AGAACAATGGAGTCCGCTGG 3′
	REV	5′ GCTGGCTGTGGATCTTCTGT 3′
Pttg1	FOW	5′ CCCTCCAACCAAAACAGCC 3′
	REV	5′ TCCCTTACCAGATTCCCATGAT 3′
Axl	FOW	5′ GTGGTTTCCAGACAACCTACG 3′
	REV	5′ CGGATGTGATACGGGGTGTG 3′
Egr1	FOW	5′ TTGTGGCCTGAACCCCTTTT 3′
	REV	5′ AGATGGGACTGCTGTCGTTG 3′
Oaf	FOW	5′ GAAGGGGCAGAGTCAGTTCC 3′
	REV	5′ GTTTTTCTGCCGGAGCTTGG 3′
Nrp2	FOW	5′ GCTGGCTACATCACTTCCCC 3′
	REV	5′ CAATCCACTCACAGTTCTGGTG 3′
Dtx3	FOW	5′ ACCCAATGTCATCACTTGGAAC 3′
	REV	5′ CCTCTTGCACCCTAGTCAGGT 3′
Mafb	FOW	5′TGGATGGCGAGCAACTACC3′
	REV	5′CCAGGTCATCGTGAGTCACA3′
Nr4a	FOW	5′TTGAGTTCGGCAAGCCTACC3′
	REV	5′GTGTACCCGTCCATGAAGGTG3′
C1qa	FOW	5′AAAGGCAATCCAGGCAATATCA3′
	REV	5′TGGTTCTGGTATGGACTCTCC3′
Bcl6	FOW	5′CCGGCACGCTAGTGATGTT3′
	REV	5′TGTCTTATGGGCTCTAAACTGCT3′
IgD	FOW	5′CTTAGCTGCCGAGAGGGATG3′
	REV	5′ACACTGTGCTCGAAGGTGTT3′
IgM	FOW	5′AACATTGCTGGCAGGGGTAG3′
	REV	5′ACCAGAGGTTGTCCCTCCTT3′
CD43	FOW	5′GACCCACTTCCTTTCCCCCT3′
	REV	5′CGTACCCAGCAAGATCATACCC3′

(*Continued*)

RT-qPCR primers	Direction	Sequence
Bcl6	FOW	5′CCGGCACGCTAGTGATGTT3′
	REV	5′TGTCTTATGGGCTCTAAACTGCT3′
Blimp1	FOW	5′TTCTCTTGGAAAAACGTGTGGG3′
	REV	5′GGAGCCGGAGCTAGACTTG3′
Traf6	FOW	5′GCACAAGTGCCCAGTTGAC3′
	REV	5′TGCAAAATTGTCGGGAAACAGT3′
EGR1	FOW	5′GGTCAGTGGCCTAGTGAGC3′
	REV	5′GTGCCGCTGAGTAAATGGGA3′
mmu-miR-146a		5′ UGAGAACUGAAUUCCAUGGGUU 3′
**Cloning primers**		
Egr1 CDS	FOW	5′AGCTAGA-AGATCT-TTCTCCAGCTCGCTGGTCC3′
	REV	5′AGCATCT-CTCGAG-TTCCTGCCTCTCCCTTTGCT3′
Egr1-3′-UTR	FOW	5′TAATCTGGTTTAAACGAGCTCTGGAAGATCTCAGAGCCAAG3′
	REV	5′TCGAATCCCTGCAGGCTCGAGGAACTTCATGTTCATAACATAC AAAAA3′
Egr1-3′-UTR-Mutant	FOW	5′P*ATGTCCACTGGACTGTCACCTC3′
	REV	5′P*GGCTGTTTCAGGCAGCTGAAG3′
EGR1-3′-UTR	FOW	5′TAATCTGGTTTAAACGAGCTCGAGGAGATGGCCATAGGAGA3′
	REV	5′TCGAATC-CCTGCAGGCTCGAGTACAAAAATCGCCGCCTACT3′
EGR1-3′-UTR-Mutant	FOW	5′P*GATGGAGCTGGACTGGAGCCAA3′
	REV	5′P*TGACCTAAGAGGAACCCTCC3′
Nrp2-3′-UTR	FOW	5′TAATCTGGTTTAAACGAGCTCACTGTGGTGGCCAAGTGAAT3′
	REV	5′TCGAATCCCTGCAGGCTCGAGCAGCACTGAGTCCCACGTTA3′
Nrp2-3′-UTR-Mutant	FOW	5′P*ACCCTTGCTGGACTGTGTATCT3′
	REV	5′P*GCGACACACACACACACACA3′
Traf6-3′-UTR	FOW	5′TAATCTGGTTTAAAGAGCTCTGAAAATCACCACTGCCTGT3′
	REV	5′TCGAATCCCTGCAGGCTCGAGGGATCCCCTCTGCTTCCTTA3′
Traf6-3′-UTR-Mutant	FOW	5′P*GGTGTTGCTGGACTGTTTAGTT3′
	REV	5′P*AGAGCGGTAACTTCTCTACTG3′
Bcl6-3′-UTR	FOW	5′TAATCTG- GTTTAAAC-GAGCTC-CCAGCCCCTTCTCAGAATC3′
	REV	5′TCGAATC-CCTGCAGG-CTCGAG CAACGCACTAATGCAGTTTAGA3′
**Genotyping primers**		
miR-146a WT	FOW	5′ CTTGGACCAGCAGTCCTCTTGATGCACCTT 3′
miR-146a KO	FOW	5′ ATCGCGGCCGCTTTAAGTGTAGAGAGGGGGTCAAGTA 3′
	REV	5′ ATTGCTCAGCGGTGCTGTCCATCTGCACGA 3′
Eμ-Myc	FOW	5′ ACCCAGGCTAAGAAGGCAAT 3′
	REV	5′ GCTCCGGGGTGTAAACAGTA 3′

Supplementary Table S2: Functional annotation results for gene expression data from miR-146a deficient tumors. Genes that were differentially expressed between tumors from Eμ-Myc 146a$^{+/+}$ and for Eμ-Myc 146a$^{-/-}$ were used as the input in the DAVID Functional Annotation Tool for Functional Annotation Analysis. Listed are keywords associated with subgroups of genes.

Term	Count	%	*P*-Value
disulfide bond	67	28.5106383	4.68E–11
signal	74	31.4893617	1.45E–10
Secreted	47	20	2.36E–10
glycoprotein	79	33.61702128	1.03E–08
innate immunity	11	4.680851064	1.82E–08
complement pathway	7	2.978723404	4.80E–07
immune response	12	5.106382979	1.23E–05
collagen	8	3.404255319	6.11E–05
transmembrane protein	15	6.382978723	1.35E–04
inflammatory response	7	2.978723404	3.21E–04
Growth factor binding	4	1.70212766	6.67E–04
chemotaxis	6	2.553191489	8.23E–04
Immunoglobulin domain	15	6.382978723	8.25E–04
inflammation	4	1.70212766	9.79E–04
extracellular matrix	10	4.255319149	9.99E–04
gpi-anchor	7	2.978723404	0.003569296
cell adhesion	12	5.106382979	0.005673897
hydroxylation	5	2.127659574	0.006928978
thiolester bond	3	1.276595745	0.007127014
immunoglobulin c region	3	1.276595745	0.007127014
sulfation	4	1.70212766	0.007435725
phosphoprotein	93	39.57446809	0.007720982
ATP	7	2.978723404	0.009174195
membrane	81	34.46808511	0.01649583
Fatty acid biosynthesis	4	1.70212766	0.01701409
cell membrane	31	13.19148936	0.018684946
tumor suppressor	5	2.127659574	0.020455856
calmodulin-binding	5	2.127659574	0.033890063
sh3 domain	7	2.978723404	0.034520031
ubl conjugation	12	5.106382979	0.047435024
lipoprotein	13	5.531914894	0.047618299
duplication	5	2.127659574	0.054099645
oxidoreductase	12	5.106382979	0.077770979

(*Continued*)

Term	Count	%	*P*-Value
thioester bond	2	0.85106383	0.079863592
Proto-oncogene	4	1.70212766	0.08178812
Pyrrolidone carboxylic acid	3	1.276595745	0.088004782
metalloprotease inhibitor	2	0.85106383	0.090742103

Supplementary Table S3: RT-qPCR analyses of individual animals for expression of B-cell maturation related antigens.

Animal	Genotype	Gender	Bcl6	Blimp1	CD43	IgM	IgD
1451	wt	f	0.271339	1.145588	0.705806	0.709198	0.177013
1454	wt	m	0.038736	0.093968	0.066144	0.558738	0.029974
1460	wt	m	8.20188	0.721423	3.902207	0.250748	0.014211
1520	wt	m	0.033797	0.174896	0.127757	0.456917	0.124269
1521	wt	m	0.135677	0.924525	0.16494	0.277375	0.037454
1523	wt	f	0.028054	0.095877	0.048391	0.195235	0.039226
1526	wt	f	0.120526	0.28323	0.324306	0.401445	0.050215
1656	wt	m	0.053368	0.108736	0.284504	0.226677	0.057909
1659	wt	m	0.161924	0.551277	0.297023	0.653097	0.130972
1749	wt	m	0.701637	3.735392	2.918962	0.750918	0.105922
1915	wt	m	0.095955	0.336614	0.960706	0.451744	0.13981
2223	wt	f	0.283138	0.463385	0.426341	0.613296	0.160568
1910	wt	f	0.030032	0.043638	0.063009	0.120665	0.021617
1655	wt	m	0.923301	0.619757	0.666354	0.397616	0.31778
1524	wt	f	1.150118	0.856506	411.4145	0.382629	2.139207
2937	wt		0.19628	0.954403	0.433843	1.408135	0.364334
2378	wt	f	0.065022	0.283746	0.193816	0.22365	0.137365
2962	wt	m	0.020592	0.077438	0.069717	0.213156	0.038049
3088	ko		0.130181	0.372912	0.349963	0.264002	0.127249
3091	ko		0.071145	0.721328	0.395792	0.820457	0.138954
1650	ko	f	24.11388	5.552898	698.29	0.549226	5.102985
1946	ko	m	19.37964	2.64538	603.3702	0.383629	3.693471
1947	ko	f	24.67962	2.261896	1709.758	0.580115	6.531063
1948	ko	f	32.45105	1.64023	1704.209	0.528875	7.289359
1954	ko	m	1.312308	2.83128	0.386467	1.10656	0.11313
2037	ko	f	16.44691	1.268977	536.9497	0.668309	5.380701
2038	ko	f	35.71832	1.959675	1262.413	0.664103	8.780564

(*Continued*)

Animal	Genotype	Gender	Bcl6	Blimp1	CD43	IgM	IgD
2222	ko	f	6.066195	1.687667	290.3511	0.311407	1.574364
1958	ko	f	0.133845	0.609388	0.570343	0.496057	0.051101
2320	ko	m	0.043995	0.332472	0.113839	0.673455	0.321443
2581	ko	m	0.044904	0.251925	0.063658	0.500618	0.190218
2590	ko	f	0.092365	0.496068	0.172707	0.88351	0.284531
2843	ko	m	0.203623	0.472634	0.582692	1.103613	0.227785
2949	ko	f	1.158255	0.482188	0.921204	0.36023	0.031063
2589	ko	m	0.070043	0.372983	0.223328	0.789975	0.045549
